# Optical coherence tomography of small intestine allograft biopsies using a handheld surgical probe

**DOI:** 10.1117/1.JBO.26.9.096008

**Published:** 2021-09-24

**Authors:** Evan T. Jelly, Jean Kwun, Robin Schmitz, Alton B. Farris, Zachary A. Steelman, Debra L. Sudan, Stuart J. Knechtle, Adam Wax

**Affiliations:** a Duke University, Department of Biomedical Engineering, Durham, North Carolina, United States; b Duke University Medical Center, Duke Transplant Center, Department of Surgery, Durham, United States; c Emory University, Department of Pathology, Atlanta, Georgia, United States

**Keywords:** optical coherence tomography, imaging systems, endoscopy, diagnostic imaging, gastroenterology

## Abstract

**Significance:** The current gold standard for monitoring small intestinal transplant (IT) rejection is endoscopic visual assessment and biopsy of suspicious lesions; however, these lesions are only superficially visualized by endoscopy. Invasive biopsies provide a coarse sampling of tissue health without depicting the true presence and extent of any pathology. Optical coherence tomography (OCT) presents a potential alternative approach with significant advantages over traditional white-light endoscopy.

**Aim:** The aim of our investigation was to evaluate OCT performance in distinguishing clinically relevant morphological features associated with IT graft failure.

**Approach:** OCT was applied to evaluate the small bowel tissues of two rhesus macaques that had undergone IT of the ileum. The traditional assessment from routine histological observation was compared with OCT captured using a handheld surgical probe during the days post-transplant and subsequently was compared with histophaology.

**Results:** The reported OCT system was capable of identifying major biological landmarks in healthy intestinal tissue. Following IT, one nonhuman primate (NHP) model suffered a severe graft ischemia, and the second NHP graft failed due to acute cellular rejection. OCT images show visual evidence of correspondence with histological signs of IT rejection.

**Conclusions:** Results suggest that OCT imaging has significant potential to reveal morphological changes associated with IT rejection and to improve patient outcomes overall.

## 1 Introduction

Intestinal transplant (IT) is a life-saving therapy for patients who are unable to absorb adequate nutrients, due to either disease or injury. The small intestine is commonly deemed one of the most challenging organs to be transplanted because of its enhanced immune response.^1^ Even with many advancements in the field, IT grafts are hypersensitive to reperfusion injury and more vulnerable to rejection, with the 10-year graft and patient survival rates still under 50% and 75%, respectively.^2^ Unlike other solid organ transplant models, there are no universally accepted biochemical markers with which to diagnose, monitor, or even predict IT graft failure.^3^ The current gold standard for post-IT evaluation involves constant and coordinated clinical, laboratory, and histopathologic testing. However, there is considerable variability over the approach and frequency of routine biopsy of acute cellular rejection (ACR) across approved IT programs in the United States.^4^ Excessive excisional biopsy of IT grafts presents several challenges, including increased financial burdens and risk of hemorrhage, perforations, and infection.^5^

OCT has shown potential utility for assessing the health of transplanted tissues at other organ sites. For example, OCT has successfully evaluated the viability of donor kidney transplants using an automated prediction algorithm.^6^ development of OCT devices specific to the small intestine has been slow, mainly due to the difficulty of performing enteroscopy across the long intraluminal distance of the small intestine, where surgical intervention and laparoscopy are commonly recommended. Post-IT, an ileostomy is performed; the small bowel is diverted through a minimal incision in the abdomen and maintained over several months to facilitate graft monitoring. This procedure reduces the distance required to be bridged by an optical probe from meters to centimeters to access the majority of the small intestine, enabling observation of the bowel endoscopically to monitor graft rejection.

OCT has become a standard of care for ophthalmic imaging and diagnostics,^7^^,^^8^ although further adoption in other tissues has been slow. While widespread use of OCT is limited in some thick tissues due to its ∼1  mm penetration depth, OCT is capable of providing micron-scale tomograms, which have proven valuable in experimental settings for imaging of coronary artery disease,^9^^–^^11^ respiratory lesions,^12^^,^^13^ breast,^14^ urinary,^15^ and reproductive^16^^,^^17^systems, among other applications. While some limitations persist, improvements in field of view (FOV),^18^ automated image analysis,^19^ image stabilization,^20^ and cost^21^ suggest that OCT will emerge as a useful technology across tissue types. Unfortunately, the penetration depth in OCT is governed by the physics of light scattering, and technical advances in penetration in highly scattering tissues are often marginal.^22^ This represents a significant burden on OCT as a medical device, whereas other tomographic imaging systems such as MRI and PET are not limited by the loss of contrast due to scattered photons. We believe that future OCT technology will rely on robust and straightforward delivery systems that directly relay the probe beam to the tissue of interest to circumvent this fundamental constraint. This avoids the problem of scattering while providing a highly targeted and precise general-purpose imaging modality to clinicians. Unfortunately, such systems have been limited, often relying on highly complex and expensive geometries involving fabricated fiber probes.^23^^,^^24^ Most OCT probes use single-mode optical fibers (SMF) to deliver the OCT signal internally, relying on a mechanical means to spin the sample arm’s distal optics to generate circumferential cross-sectional images.^25^^–^^29^ These devices are often complex and expensive, are not built as general-purpose probes, and are still nascent as medical devices.

For OCT probes, the gastrointestinal (GI) tract is a particular area of focus, where previous efforts have made significant progress evaluating or diagnosing conditions, such as Barrett’s esophagus,^30^ esophageal cancer,^31^ inflammatory bowel disease,^32^ and ulcerative colitis.^33^ However, comparatively few OCT studies have been reported in the small intestine. Partly due to its elongated and sinuous structure and the significant intraluminal distance from either the oral or anal orifices, the small intestine remains difficult to image even with established endoscopic modalities.

Previous OCT of the small intestine often originated as extensions of studies of the stomach, where long GI endoscopes were capable of accessing the pylorus, which marks the region between the stomach and the duodenum.^17^ Hsiung et al. were among the first to image the small intestine using OCT, imaging excised surgical samples and comparing them against corresponding histology.^34^ *In vivo* imaging of the terminal ends of the small intestine has since been realized^35^^,^^36^ and shows that OCT can observe structures such as villi and submucosa.^37^^,^^38^ The clinical relevance of this information can be seen in studies in which OCT has been directly used to diagnose celiac disease in children, reporting sensitivity and specificity of 82% and 100% against histology, respectively, between healthy and atrophic villous structures.^39^ These results illustrate the diagnostic potential for OCT in the small intestine.

We have previously reported the endoscopic delivery of OCT using a handheld rigid borescope^40^ and a custom, portable spectral domain OCT (SD-OCT) system shown to offer comparable imaging performance to commercially available systems.^41^^,^^42^ The potential utility of this device was first validated as a method to measure the thickness of knee cartilage for cases of severe osteoarthritis. We now demonstrate the use of this borescope-based OCT system to monitor transplanted small intestinal tissue in a nonhuman primate (NHP) model.

The handheld borescope-based OCT instrument was validated in studies of two rhesus macaques that underwent IT. Progression of transplant allograft rejection was assessed using surveillance surgical biopsies with the results from OCT analysis compared with H&E-stained slides from coregistered biopsies. Our results suggest the potential for OCT to identify morphological features indicative of graft rejection in the small bowel and contribute to the development of transplant monitoring strategies that can facilitate long-term intestinal graft function.

## 2 Methods

### 2.1 Endoscopic OCT Instrumentation

A compact, portable spectral-domain OCT (SD-OCT) engine, first reported by Kim et al.,^41^ was used for these studies. The system features a high-powered broadband superluminescent diode (Exalos) with an 840-nm center wavelength and 48-nm full-width at half-maximum (FWHM) bandwidth. To mitigate unwanted intensity fluctuations caused from using an uncooled source, constant background acquisition and subtraction were applied in processing. A custom spectrometer with high spectral resolution (<0.1  nm) that utilized a tall-pixel CMOS line array to reduced sensitivity to temperature fluctuations and mechanical stress was used. Combining these elements yielded a theoretical 2.8-mm imaging depth in air and coherence-limited axial resolution of 7  μm. An onboard small-form-factor (SFF) computer (Intel) coordinated frame acquisition and beam scanning in the OCT probe, rendering (512×512  pixels) B-scans at a rate of 12 frames per second.

**Fig. 1:**
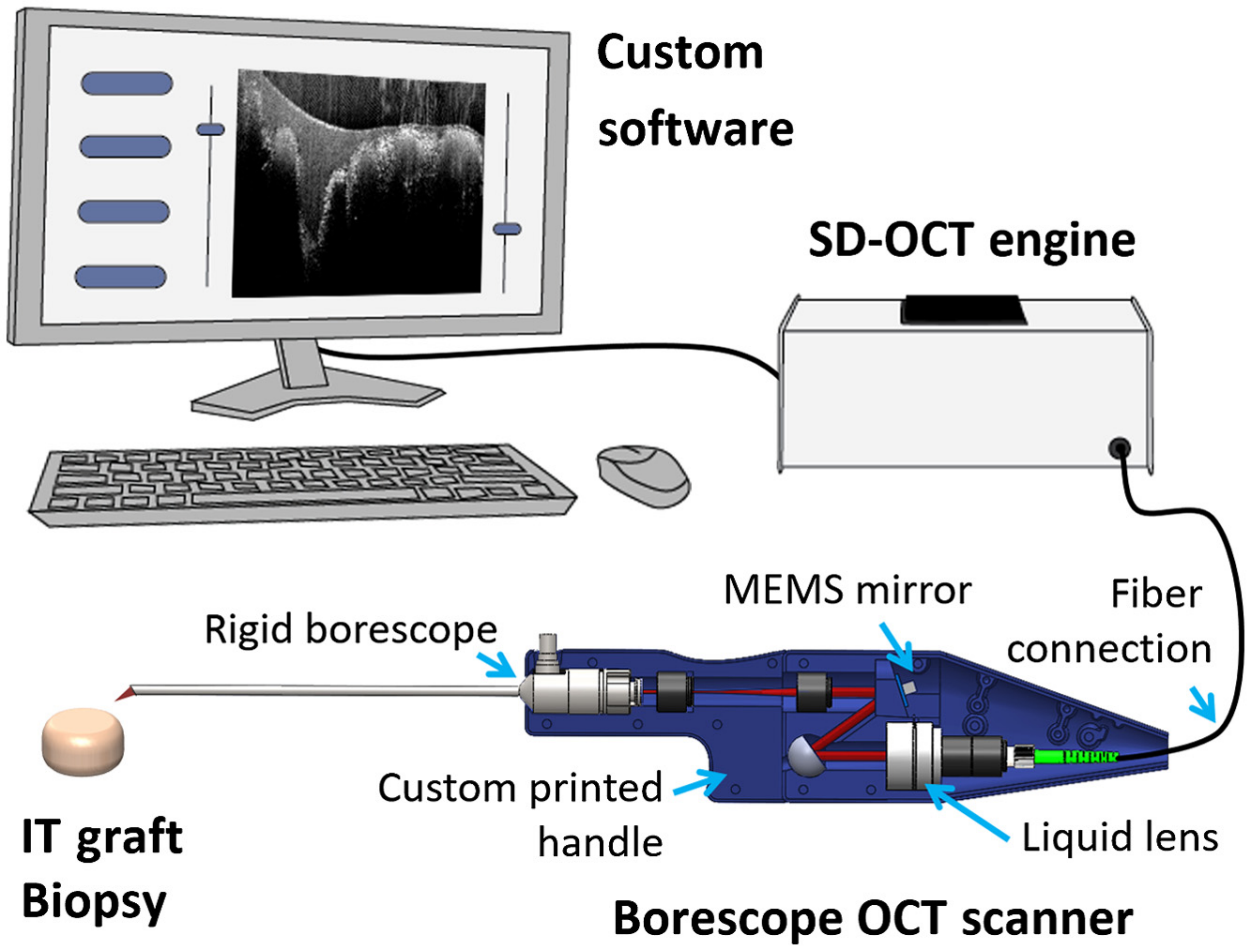
Schematic illustration of Borescope OCT system. Custom GUI software control an SD-OCT engine: 840±20  nm broadband light source, custom-built spectrometer, and SFF PC. The instrument’s sample scanning mechanisms coupled NIR light, delivered by SMF, to a handheld 30-deg-angled rigid borescope.

We have previously adapted this OCT engine for endoscopic use by adapting a commercial 30 cm rigid borescope with a 4 mm diameter.^40^ In this study, a prototype 30-deg-angled rigid borescope (Foreal Spectrum) treated with an antireflective coating near 840 nm was employed. The distal profile of the borescope was 4 mm in diameter with a 20-cm long probe. Figure 1 shows the instrument schematic. The fiber optic output from the OCT engine enclosure was collimated using a 10-mm liquid lens (Optotune) and incident on a 3.6-mm wide microelectromechanical system (MEMS) mirror (Mirrorcle). A 4f lens relay produced a scan pattern at the input image plane of the borescope that was then relayed, via a series of internal small relay lenses, to the sample plane at the distal end of the probe. The liquid lens allowed for electronic control of the beam focus for a variable working distance of 0 to 2 mm. The resulting design produced a scanning field of ∼±0.6  mm about the center axis of the borescope’s back aperture, which yielded a distal scan area of 2.5  mm×2.5  mm after the borescope. Custom software, written in C#, displayed real-time B-scans and allowed for user adjustments to the focal plane, numerical dispersion correction,^43^ and sample scan pattern.

### 2.2 OCT Borescope

The first iteration of a borescope OCT device featured a 30-cm long flat-angled borescope.^40^ However, many endoscopic applications require a low-angle direction of view (DOV) to adequately compensate for unique approach angles between the surgical entry port and operative site. For this study, a 30-deg DOV borescope was used to realize our design’s clinical potential further. Scanning of the OCT beam was limited to one lateral dimension to avoid nonsymmetric group-velocity dispersion introduced from a nonuniform pathlength across the acute axis of the borescope’s 30-deg wedge prism. A 24.46% loss of power was observed between the scanner body and the borescope output due to the high number of air-spaced relay lenses within the borescope. In this study, the length of the borescope used was 10 cm less than in our previous work, delivering 2.12 mW at the sample and 92-dB signal-to-noise (SNR) and improving overall transmissivity and sensitivity by 8% and 5.7%, respectively. Axial resolution was measured at 8.2  μm, using the FWHM of the A-line peak of a mirror located at the borescope’s focal plane and within <1% of the theoretical axial resolution of 8.13  μm. The lateral spot size of the system was 10.3  μm FWHM across the 2.5  mm×2.5  mm FOV, as measured using a CMOS camera (Flea3, FLIR, 4.8  μm pixels). For the minimum observed spot size of 10.3  μm, power at the sample remained below the maximum permissible exposure for skin established by the American National Standards Institute (ANSI Z136.1).

### 2.3 Animal IT Model

Animal subjects ranging from 4 to 6 kg were purchased from Alpha Genesis (Yemassee, South Carolina). Two male rhesus macaques, hereinafter referred to as subjects 1 and 2, underwent orthotopic IT with the Abdominal Transplant Group at Duke University. Donor–recipient pairs were maximally major histocompatibility complex classes I and II mismatched. Animal subjects did not receive any immunosuppressive treatments to study unmanipulated rejection in allogeneic IT. Intestinal biopsies were performed on days 0, 2, and 6 after transplant from a defunctionalized limb of proximal jejunum via partial laparotomy. In addition, optical coherence tomography (OCT) of each biopsy was utilized for minimally invasive surveillance of the graft. The study endpoint was defined as clinical evidence of acute rejection with intestinal graft failure. All animals were housed in accordance with the National Institutes of Health (NIH) guidelines and were approved by the Duke Institutional Animal Care and Use Committee (IACUC number: 087-19-04).

### 2.4 NHP OCT Imaging Protocol

OCT imaging for this study was performed coinciding with surgical observation of NHP with IT grafts. Imaging technicians in surgical protective attire were presented with *ex vivo* IT allograft surgical specimens immediately following removal. The working distance of the scanning probe was set to ∼1  mm to provide contactless imaging of the biopsy. Handheld operation of the scanning probe allowed for unrestricted movement of the probe and rapid adjustments and translation between regions of interest. Operators securely held the base of the scanner body while placing a second hand near the distal-end of the probe to increase stability to allow for finer manipulation of the imaging field. OCT B-scans were acquired across the entire volume of the biopsy. B-scans were captured at a rate of 12 fps and compiled into 10-frame averages in postprocessing. After OCT imaging, IT graft samples were preserved for later histological analysis.

### 2.5 Histology

Intestinal graft tissue biopsies were fixed with 10% neutral buffered formalin and submitted for processing into paraffin blocks. Paraffin blocks underwent serial sectioning (5  μm) and staining with hematoxylin and eosin (H&E) for histological evaluation and rejection grading. Whole slides of graft samples were scanned with an Aperio ScanScope XT (Aperio Technologies, Inc., Vista, California). Allograft histology was evaluated in a blinded fashion by a trained transplant pathologist (A.B.F).

## 3 Results

### 3.1 Comparison of Jejunum and Ileum

**Fig. 2:**
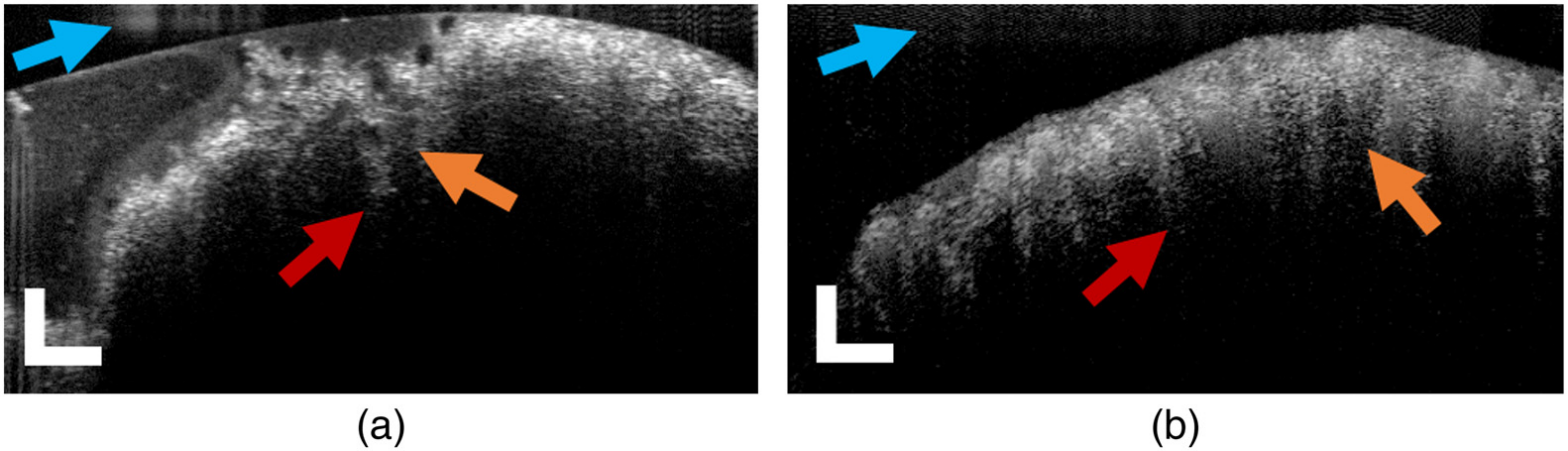
Optical coherence tomography of the small intestine performed using a handheld surgical probe. (a) *Ex vivo* OCT B-scans of an NHP jejunum biopsy. (b) *Ex vivo* OCT B-scans of an NHP ilium biopsy. Examples of intestinal crypts and villus structures are identified by red and orange arrows, respectively. Blue 1 arrows indicate mirror artifacts resulting from internal lenses of borescope. 10-frame average; scale bars=250  μm.

*Ex vivo* jejunum and ileum samples were imaged with the 30-cm flat-angled handheld OCT probe immediately after tissue harvest. Representative OCT images of the healthy NHP jejunum and ileum are shown in Figs. 2(a) and 2(b), respectively. The general structures of the jejunum and ileum of the rhesus macaque are comparable, as the intestine’s mucosal lining is fairly uniform throughout its entire length.^44^ Images of both the jejunum and ileum clearly display the characteristic striping features of intestinal villi. The epithelial cell layer is also visible, appearing as a thin scattering band separating an outer layer of mucus from individual villi.

Morphological differences were noted across image sets from the jejunum and ileum upon inspection. The shape of villi can vary in form-factor across the length of the small intestine, ranging from narrow finger-like structures to broader leaf-like forms.^45^ The mean and standard deviation of villi thickness across both samples were measured manually from five randomly selected B-scans for each sample using ImageJ. Thicknesses varied substantially in 26 total measurements for each sample. In general, the jejunum exhibited thicker villi (146±60  μm), whereas the ileum exhibited thinner villi (108±22  μm). This result is expected as a higher density of villi is observed near the ileum terminus.^45^ Further, a paired t test comparing matched OCT-derived villi thicknesses with measurements made from corresponding H&E images returned a p value of 0.63, indicating no significant difference in the means observed between the two techniques.

### 3.2 Identification of Intestinal Landmarks

**Fig. 3:**
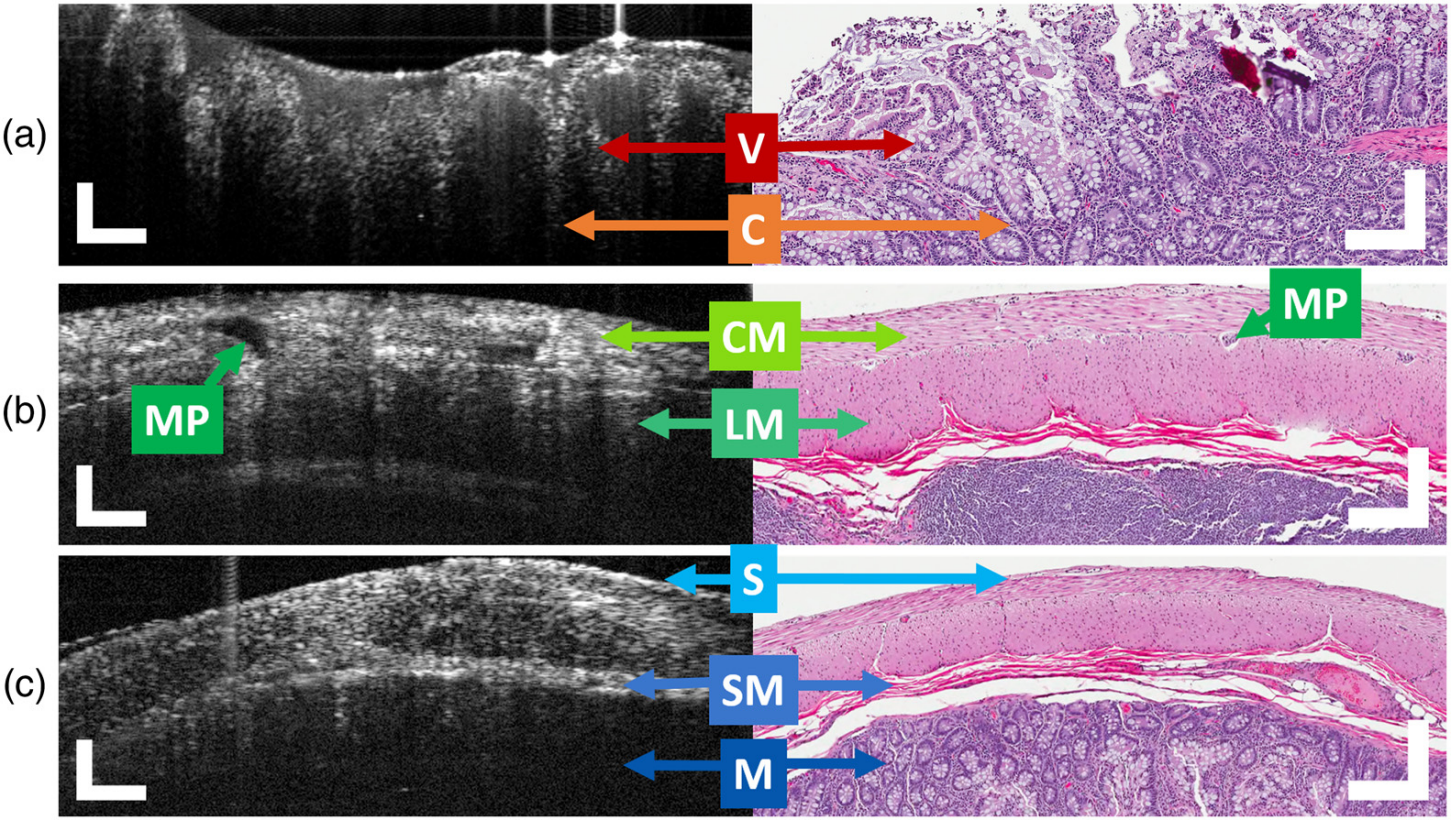
Representative cross-sectional imaging of *ex vivo* NHP ileum using OCT and histology. (a) An interior projection OCT B-scan (left) and corresponding histology (right) identifying a villus (V) and crypts (C) of healthy intestinal mucosa. (b), (c) Two exterior projection OCT B-scans (left) and corresponding histology (right) identifying several structural layers: circular smooth muscle (CM), myenteric plexus (MP), longitudinal 1 smooth muscle (LM), serous membrane (S), submucosa (SM), and mucosa (M). OCT images are 10-frame averages. Scale bars=250  μm.

Figure 3 shows 10-frame averaged OCT B-scans captured using the handheld 30-deg angled DOV borescope OCT device against H&E micrographs from the corresponding sample. Images were acquired from normal tissue samples harvested prior to transplant from an NHP-IT surgical study. The 5.7% improvement in SNR in OCT images from previous designs is visually apparent when compared with images in Fig. 2. The borescope provides good contrast by reducing unwanted background intensity due to a reduction in optical elements in the borescope’s relay arm; this feature reduces the effect from optical surface reflections and internal etaloning overall. OCT images captured in this configuration also show no signs of nonuniform field distortion resulting from scanning along the borescope’s angled prism.

Throughout the length of the small intestine, the epithelial layer is identified by two major morphological features: villi and crypt epithelium. As previously discussed, villi are presented as long narrow projections that protrude into the intestinal lumen; crypts, or intestinal glands, are moat-like invaginations of the lower 20% of the epithelium and are the site of epithelial cell division.^46^ Two interior projection OCT B-scans are presented with comparative H&E images in Fig. 3(a). We identified healthy mucosal structures on the OCT images, including a clear demarcation between the outer mucus and lamina propria separated by the higher-scattering epithelial cell layer of healthy villus. Further, OCT depth penetration was sufficient for visualizing the beginning of the crypt region of the sample.

**Fig. 4:**
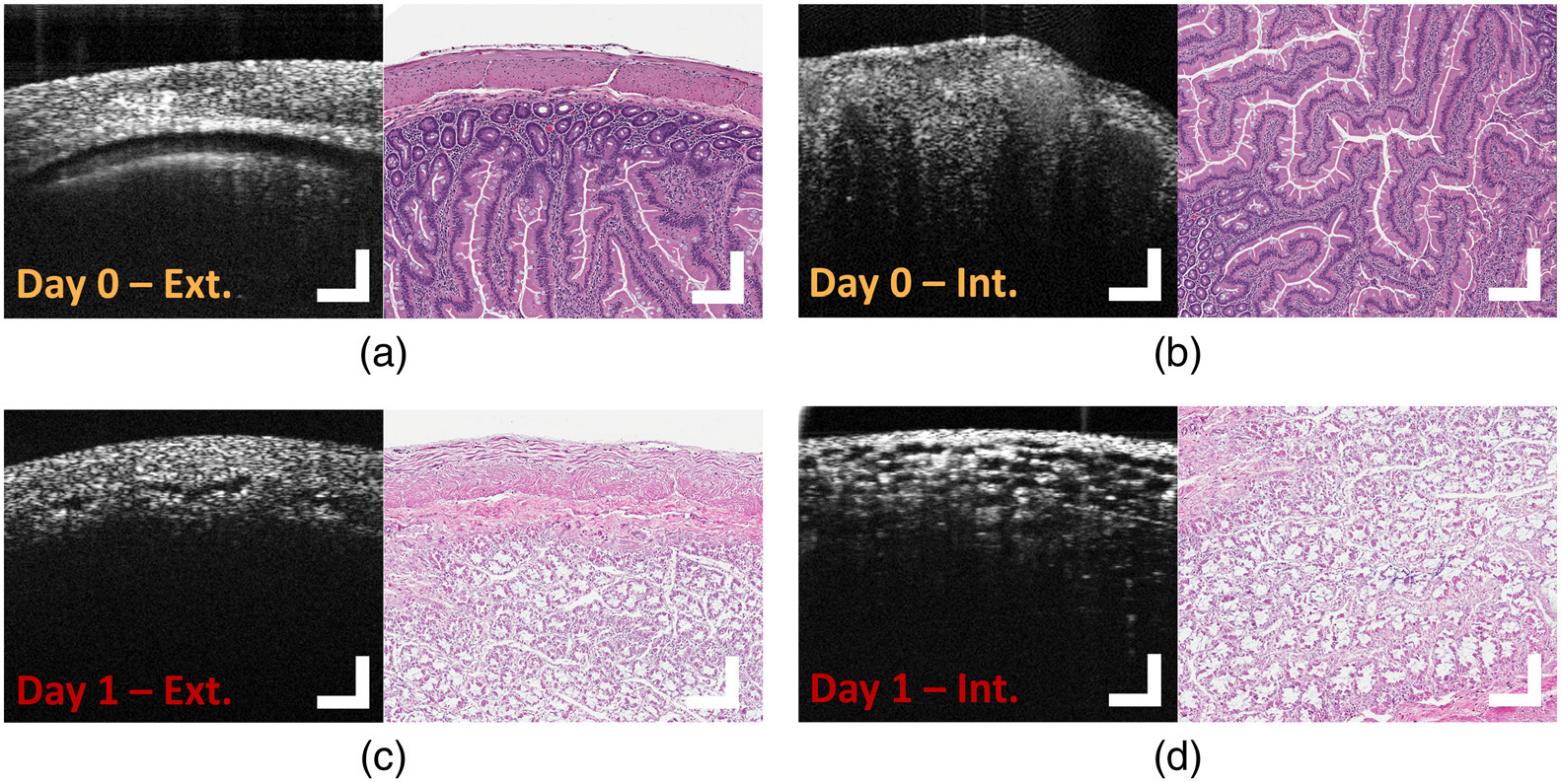
OCT B-scans (left) and corresponding histology (right) images of NHP ileum IT allograft ischemia. (a) External and (b) internal projections of excised samples of normal IT allografts observed at day of transplantation. (c) External and (d) internal projection of excised samples of highly rejected IT allografts observed one day after IT. OCT images are 10-frame averages; scale bars=250  μm.

Several layers of specialized tissue make up the GI wall surrounding the lumen of the small intestine: serous membrane, muscularis propria, submucosa, and mucosa. These layers are identified in Figs. 3(b) and 3(c). The muscularis propria consists of two primary types of smooth muscle, longitudinal and circular, working in tandem to mediate peristalsis. OCT images acquired from the exterior of the small intestine exhibit distinct layers resembling the same morphology as the boundaries between the inner and outer muscular layers seen in corresponding H&E [Fig. 3(b)]. Further, the myenteric plexus (Auerbach’s plexus), identified by a dense clustering of ganglion cells, can be observed between these two muscle types. Several features can be seen in the OCT images of the muscularis propria, which correlate well with these features. Layers of the muscularis propria range in size distribution across images. In Fig. 4(b), the outer muscular layer thickness is reduced, yielding improved contrast of shallower layers. The serous membrane, the outermost layer of small intestine consisting of several thin layers of mesothelium, is observed in both representative images from OCT imaging and histology. The connective tissue framework of the submucosa, as well as the beginning of the mucosal layer, can also be clearly identified in both sets.

### 3.3 Evaluation of IT Graft Ischemia (Subject 1)

Observations of ITs in this study were guided by the dynamics of transplant failure in our NHP model. Immediately following transplantation, subject 1 suffered a thrombosis of the artery, leading to graft ischemia prior to the scheduled imaging on the day following transplantation. Surgical samples of the failed IT allograft were removed immediately following sacrifice on day 1 and presented to OCT imaging technicians within a half hour of tissue harvest. Example OCT images from subject 1 allograft captured both on the initial day of transplantation and prior to expiry are presented in Fig. 4. OCT images of healthy allograft samples from subject 1 [Figs. 4(a) and 4(b)] maintain a high degree of contrast between all previously identified layers of both the exterior GI wall and lumen, showing no indication of pathology. OCT images from ischemic IT tissue 24 h post-transplant [Figs. 4(b) and 4(c)] present stark comparison with healthy tissue. Figure 4(c) shows a blurring of internal structures and a strong reduction in optical scattering of the exterior GI wall. These are strong indications of changes in the intracellular morphology and consistent with reports of apoptotic tissue damage imaged using OCT.^47^ Images of the same sample from an internal projection of the lumen [Fig. 4(d)] reveal a frequency of sponge-like structures observed throughout the rejected tissue samples, resembling severely edematous tissue imaged using OCT.^48^

### 3.4 Evaluation of IT Graft Rejection (Subject 2)

**Fig. 5:**
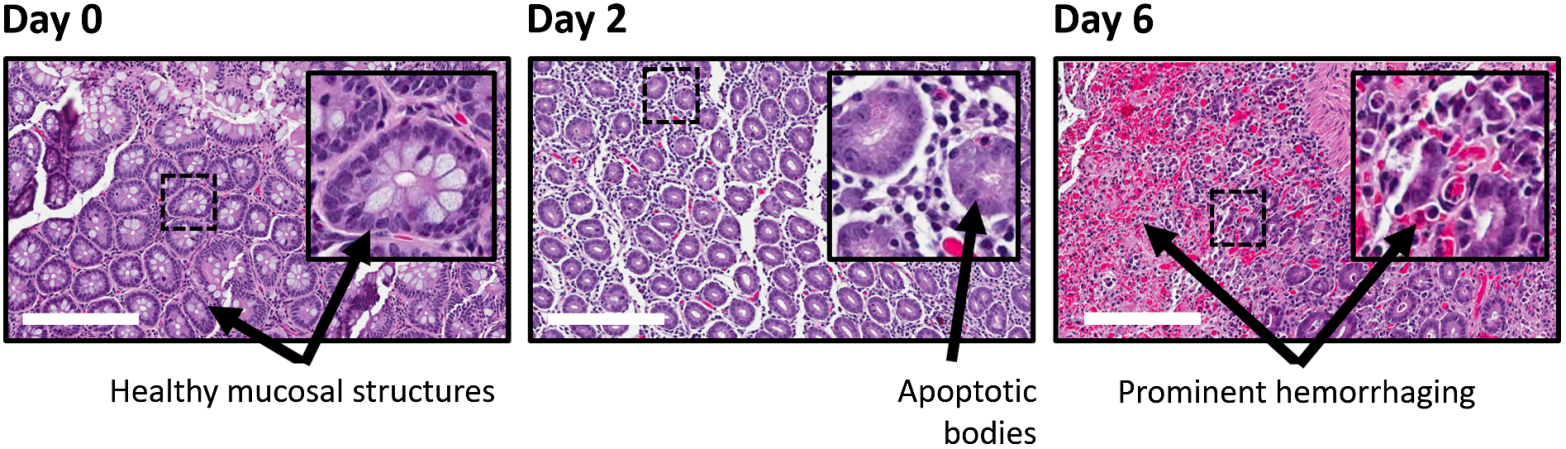
Cellular biomarkers of ACR in IT grafts. H&E-stained NHP allograft specimens of the small intestine on days 0, 2, and 6 after transplantation. Insets: 85×85  μm FOV with corresponding location marked by a dashed line. Scale bars=250  μm.

For subject 2, the small bowel rejection occurred gradually, allowing for evaluation of tissue pathology over the full 7-day trial. Analysis of fixed and sectioned biopsy samples under H&E staining by IT pathology experts at Emory University was utilized to visualize the tissue alterations and gave guidance for identifying structures using OCT. At day 0, the intestinal mucosa showed no diagnostic abnormality. At day 2 after transplantation, the graft intestinal mucosa showed slight villous blunting, increased intraepithelial lymphocytes, and an occasional neutrophilic crypt abscess. Relatively sparse apoptotic bodies were present. Overall, the findings were compatible with mild to moderate (grades 1 to 2) ACR, and the neutrophilic crypt abscess could represent the residual effects of reperfusion injury. At day 6 after transplantation, the graft intestinal mucosa showed marked injury with a total loss of glandular bodies or “crypt dropout,” epithelial sloughing, lamina propria hemorrhage, and foci of frank ulceration. In areas of preserved epithelium, scattered apoptotic bodies could also be seen, and there was a patchy increase in intraepithelial lymphocytes. Graft findings overall were compatible with severe (grade 3) ACR.^49^ The key biomarkers of ACR identified by histological analysis are presented in Fig. 5.

**Fig. 6:**
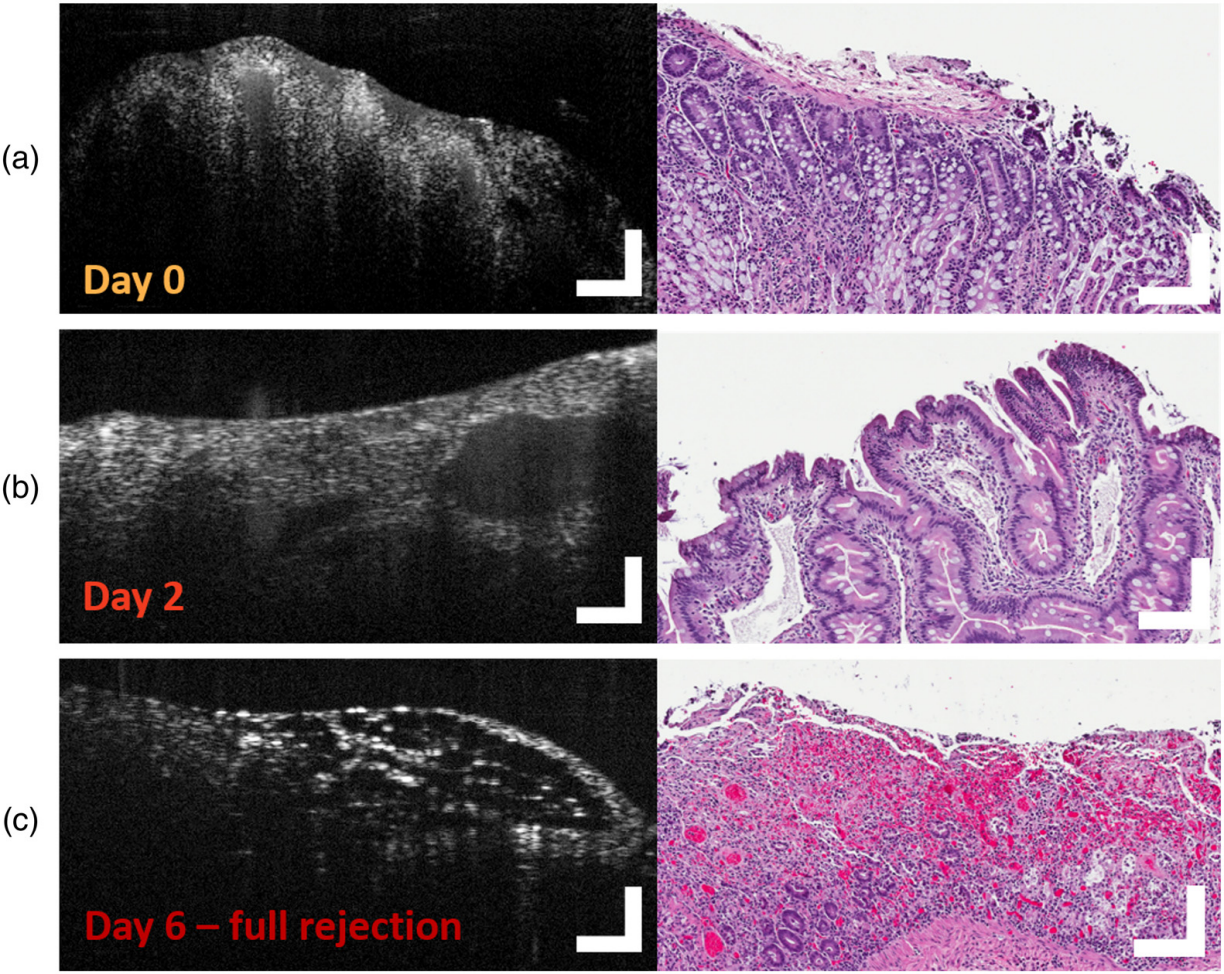
Progression of ACR in IT grafts from an NHP model. OCT B-scans (left) and corresponding histology (right) of surgically excised IT allografts imaged 0, 2, and 6 days post IT visualizing (a) negative, (b) mild to moderate, and (c) severe grades of ACR. 10-frame average OCT images; scale bars=250  μm.

Apparent changes in tissue composition were observed upon review of OCT images from subject 2 IT specimens. Example OCT images from subject 2 allografts, presented as Fig. 6, illustrate several biomarkers of transplant rejection. At day 0, the initial day of the transplant, there was strong evidence of healthy mucosal structures in the OCT images [Fig. 6(a)]. Large features with greatly reduced scattering are present at day 2 (grades 1 to 2 ACR). These features are likely indicative of lamina propria inflammation and diffuse epithelial reactive changes in crypts identified by H&E. This crypt dropout can be confirmed by OCT. A decrease in overall scattering may be the result of abundant and focally confluent crypt apoptotic bodies [Fig. 6(b)]. At day 6 (grade 3 ACR), OCT images exhibit signs of full rejection. Prominent ulcerations of the muscularis propria were identified by OCT. Further, the structure of rejected tissue is dominated by sponge-like infarcts as well as significant changes in the scattering properties of the sample. This morphology is mirrored in frequency by inflammation within corresponding H&E micrographs indicated by neutrophilic infiltration and increased intraepithelial lymphocytes throughout the sample [Fig. 6(c)].

## 4 Discussion

OCT is presented as a modality for evaluating the status of transplanted intestinal tissue. The utility of a handheld rigid endoscopic probe demonstrates the potential to fit into the clinical workflow for graft monitoring. The rigid borescope enables access to transplanted tissue in patients using a temporary ostomy during the critical early weeks post-transplantation. The use of the 30-deg DOV borescope applied here further advances the technology by better adapting it for clinical imaging, while also improving throughput and SNR. Future work may involve combined OCT and traditional endoscopic visual or fluorescence imaging through the addition of a white-light light source and camera fitted to the borescope’s auxiliary port. The combination will allow for coregistration of the white-light images with OCT-imaged tissue sites. Continued studies will also look to provide analysis of histological structure with quantitative analysis of *in vivo* OCT images. Given the expected range of tissue status, these data will be very useful for developing biomarkers in the NHP-IT model.

ACR is the leading cause of post-operative IT graft loss, with most patients experiencing at least one episode.^50^ There are many indications of ACR in IT patients; however, there are no universally accepted markers to predict rejection. Numerous chemical biomarkers have been investigated to predict and even exclude rejection, including citrulline levels and cytokine profiles in the peripheral blood, stool calprotectin levels, and changes in various transcriptome sets.^51^^–^^53^ However, none have shown the required sensitivity, reproducibility, or turnaround speeds required to be universally recommended for post-transplant evaluation.^54^ Across all published grading schemes, apoptotic body count (ABC) in crypt cells is identified as a primary indicator of onset ACR. Changes in epithelial morphology, density, and composition, which occur due to apoptosis in crypt bodies, will alter optical scattering properties, which can be visualized using OCT.

Epithelial injury and inflammation in the lamina propria are important correlates for identifying allograft rejection.^55^ In many low ABC cases, increased mononuclear inflammation and crypt injury within the allograft are predictive of graft rejection.^56^ However, increased inflammation in the lamina propria is most easily diagnosed using endoscopic data and may not be easily observable by H&E during the initial months post-transplantation when early detection is critical. OCT images of intermediate stage ACR were dominated by large homogeneous regions in the normal mucosal structure, identified as lamina propria inflammation when correlated to expert H&E analysis. The evidence presented supports OCT being able to provide real-time identification of morphological features indicative of ACR.

Using current methods, it is essential that gross endoscopic clinical findings are confirmed against histopathologic biopsy, as endoscopic visual inspection alone provides insufficient sensitivity and specificity to diagnose ACR.^57^ The ileum is subject to the highest degree of acute rejection, as it maintains the highest concentration of lymphoid tissue in the gut, and therefore requires intense surveillance. For this reason, IT centers conduct two allograft biopsies per week for the first 6 weeks post-transplant and gradually decrease to once every 3 weeks, even in the absence of any symptoms of graft dysfunction.^4^ Since rejection may be focal, a minimum of three biopsies of the IT graft are often recommended, with sufficient depth to include observation of the full mucosal layer, muscularis mucosae, and superficial submucosa.^56^ Integrating OCT into the post-IT clinical workflow adds a much-needed layer of confidence toward identifying suspicious lesions, greatly reducing the number of biopsies required to diagnose and stage ACR accurately.

An important limitation of our system is the modest acquisition speed of the OCT engine (12 B-scans per second), which resulted in moderate motion artifacts in averaged B-scans. Two-handed operation of the OCT scanner was required to stabilize the distal end, which diminished motion artifacts. In preparation for *in vivo* work, future iterations of our probe will incorporate faster scanning speeds, along with advanced image registration techniques to address these concerns.

## 5 Conclusion

We have presented the application of an endoscopic tool for assessing the status of transplanted intestinal tissue. The approach, based on a compact, portable OCT engine, has been adapted for endoscopic use using a rigid borescope and offers a form factor that will facilitate use in the operating suite. OCT is uniquely positioned as an informative imaging tool to assist in evaluating and grading IT rejection. OCT shows a promising path for *in vivo* analysis of IT-related pathologies by providing high-resolution cross-sectional images of the GI wall of the small intestine. We hope that this work further promotes OCT applications in the small bowel and improved understanding of the clinical progression of IT-related pathologies.
